# Mechanical Circulatory Support for Single Ventricle Failure

**DOI:** 10.3389/fcvm.2018.00115

**Published:** 2018-08-28

**Authors:** Massimo Griselli, Raina Sinha, Subin Jang, Gianluigi Perri, Iki Adachi

**Affiliations:** ^1^Division of Pediatric Cardiac Surgery, Department of Surgery, University of Minnesota Masonic Children's Hospital, Minneapolis, MN, United States; ^2^Fondazione Policlinico Universitario A. Gemelli, Università Cattolica del Sacro Cuore, Rome, Italy; ^3^Division of Congenital Heart Surgery, Michael E. DeBakey Department of Surgery, Texas Children's Hospital, Baylor College of Medicine, Houston, TX, United States

**Keywords:** single ventricle, mechanical circulatory support, fontan physiology, ventricular assist devices, VA ECMO

## Abstract

Mechanical circulatory support (MCS) for failing single ventricle (SV) physiology is a complex and challenging problem, which has not yet been satisfactorily addressed. Advancements in surgical strategies and techniques along with intensive care management have substantially improved the outcomes of neonatal palliation for SV physiology, particularly for hypoplastic left heart syndrome (HLHS). This is associated with a steady increase in the number of SV patients who are susceptible to develop heart failure (HF) and would potentially require MCS at a certain stage in their palliation. We have reviewed the literature regarding the reported modalities of MCS use in the management of SV patients. This includes analysis of various devices and strategies used for failing circulation at distinct stages of the SV pathway: after neonatal palliation, after the superior cavo-pulmonary connection (SCPC), and after total cavo-pulmonary connection (TCPC).

## Introduction

Fontan and Baudet introduced the concept of single ventricle (SV) palliation in 1968, in a patient diagnosed with tricuspid atresia, by total right heart bypass achieved with atrio-pulmonary connection (1). SV palliation is considered for patients with severe congenital heart disease (CHD) that are not amenable to biventricular repair. The primary aim of SV palliation is to obtain separation of left and right circulations, which would allow oxygenated blood flow to the systemic circulation, while deoxygenated blood is directed to the pulmonary circulation, without contribution from a sub-pulmonary ventricle (2–4). The Fontan palliation, including total cavo-pulmonary connection (TCPC), produces a unique physiology in which pulmonary blood flow is driven primarily by the residual force of the systemic ventricle, resulting in an elevation in systemic venous pressure. Although, there have been substantial improvements in early to mid-term outcomes owing to the several surgical modifications of the original technique, long-term morbidities inherently associated with the peculiar Fontan circulation remain a challenge (5–7). Long-term complications are due to the inevitable systemic venous hypertension and low cardiac output state, thus there has been a pessimistic view that all “Fontans” will fail at some point. In a recent analysis of data from multiple children's hospitals in the United States, approximately 12% of all hospitalizations of children with SV were complicated by heart failure (HF) (8). Given the shortage of cardiac allografts, it is obvious that cardiac transplantation alone is not a sustainable solution to address the epidemic of HF associated with SV physiology. Hence, the need for alternative options, particularly mechanical circulatory support (MCS), has been increasingly recognized.

## Mechanical circulatory support type

As reported in previous studies (9–11), patients with SV physiology are subject to drastic alterations of their clinical condition over time. The concept of MCS in this group of patients is based on the need to reduce their systemic venous pressure while augmenting cardiac output. Most patients are poor transplantation candidates because of a wasted nutritional status and multiple organ dysfunction. The implantation of a cardiac assist device may reverse organ dysfunction and replenish these children but the experience in use of ventricular assist devices (VADs) in univentricular hearts remains a challenge. Three main support types are available for assisting failure of SV physiology: a veno-arterial extracorporeal membrane oxygenator (VA-ECMO) used for short term cardiac support, a ventricular assist device (VAD) that may be used for mid and long-term support, bridge to recovery and typically bridge to transplant, and the Total Artificial Heart (TAH). Table 1 lists the MCS modalities along with their advantages and disadvantages.

**Table 1:** Most common MCS Devices for SV.

**Types**	**Advantages**	**Disadvantages**	**Size**
VA-ECMO (continuous flow)	Immediate establishment	Finite use Anticoagulation and transfusion requirement Cost of hospital stay	
VAD	Possibility of extubation Discharge from acute setting Allowance of longer waiting time Reduced anticoagulant requirement Better (than VA-ECMO) ventricular recovery Decreased use of blood products and cost	Limitation of patient body size	
* Centrifugal VADs* (continuous flow) CentriMag RotaFlow	Short term MCS Relative ease of implantation Adaptable to all ages Ability to convert into VA-ECMO by inserting an oxygenator Provide renal support with HD		
* Berlin Heart Excor* (BHE) (pulsatile)	Ability to use as UVAD or BiVAD for SV		10–15–25–30–50–60–80 ml
*Intracorporeal VAD* (continuous flow) HeartMate I, II (Axial flow) HeartWare, HeartMate III (Centrifugal pumps)			
TAH • SynCardia	•BiV support •Can be used for SV failure	Unsuitable for smallest pediatric patient	50cc, 70cc

*VA-ECMO, veno-arterial extracorporeal membrane oxygenation; VAD, ventricular assist device; MCS, mechanical circulatory support; HD, hemodialysis; SV, single ventricle; TAH, total artificial heart; BiV, biventricular support*.

VA-ECMO is the most common type of MCS used in children, not only because it can provide biventricular cardiac and respiratory support, but also due to lack of alternative technology suitable for the pediatric population. In the setting of acute cardiac failure, VA-ECMO support remains an important option because it can be established immediately and maintained for a period of duration until correctable factors are identified. It may be used as bridge to decision or bridge to a more long-term device or to transplantation. In essence, VA-ECMO is a closed cardiopulmonary bypass system with the venous drainage cannula in jugular or femoral vein or right atrium. The blood is pumped through a membrane oxygenator with heat exchanger for temperature regulation and returns to the patient via an aortic cannula in the carotid or femoral artery or ascending aorta.

Recently, technological advancements and experience have improved the durability and decreased the complication profile of VADs, with a consequent 10-fold increase in the number of VAD implantations as reported by the Interagency Registry for Mechanically Assisted Circulatory Support (INTERMACS) from 2006 to 2010 (12). In cases of SV with ventricular dysfunction without respiratory failure, VADs have been shown to be efficacious compared with VA-ECMO, due to reduced anticoagulant and transfusion requirements, earlier ventricular recovery, and decreased cost (13). Additional advantages of a VAD include the “possibility of extubation, discharge to a semi-ambulatory high-dependency setting, and allowance of longer waiting times for an organ” (14). This is crucial for SV patients who have long transplant waiting times, owing to patient size as well as a high incidence of pre-formed alloantibodies (14, 15). Since worldwide experience in supporting these patients is limited, it is unclear which device would afford the best chance of survival, considering the complex pathophysiology of SV failure (9).

A short-term MCS employed in many centers are centrifugal VADs, which have gained popularity as bridge to decision or toward long-term VADs or to transplantation. They are relatively easy to implant and can be adapted to all age groups. The Levitronix CentriMag (Levitronix GmbH, Zurich, Switzerland) and the RotaFlow from Maquet (Maquet Medical System, Wayne, New Jersey) are most frequently used in the United States. They can be supplemented with an oxygenator to simulate a VA-ECMO and provide renal support with a dialysis machine (16).

However, the most common VAD used amongst the pediatric population is the Berlin Heart Excor (BHE) (Berlin Heart Inc, The Woodlands, TX). The BHE consists of a para-corporeal, pneumatically driven, polyurethane blood pump (10, 15, 25, 30, 50, 60, and 80 ml) (17) with a multilayer flexible membrane separating blood from the air chamber. Silicon cannulae connect the blood pump to the patient, and tri-leaflet inflow and outflow valves prevent blood reflux. All the surfaces in contact with blood are heparin-coated. Each pump is driven by a pulsatile electro-pneumatic system. The drive unit (IKUS 2000) has a triple operational control and pneumatic system, with the availability of synchronous and asynchronous operating models. In SV failure, the BHE has been utilized in vast majority of cases as a univentricular assist device (UVAD), where it supports the SV exclusively, leaving the systemic-to-pulmonary shunt or the superior cavo-pulmonary connection (SCPC), or the total cavo-pulmonary connection (TCPC) as unassisted sources of pulmonary blood flow. The BHE has been used as “biventricular support” or a BiVAD in very few cases, where the systemic and pulmonary circulations have been isolated and supported individually (18).

The last category of VADs used are intracorporeal devices. These continuous flow pumps can be differentiated into axial flow (HeartMate I and II, Thoratec Corp, Pleasanton, CA) or centrifugal pumps (HeartWare HVAD, HeartWare Inc, Framingham, MA and HeartMate III, Thoratec Corp, Pleasanton, CA), with different flow rates and shut off pressure thresholds. There has been considerable discussion pertaining to their individual unloading characteristics as well as comparison with pulsatile flow pumps, when accounting for the mechanism and timing of SV failure. Horne et al. have described a useful algorithm for the treatment of SV failure in various age groups, with respect to the aforementioned devices' specifications, size of patient, and underlying mechanism (9).

The remaining MCS device available is the Total Artificial Heart (TAH) by SynCardia (SynCardia, Tucson, AZ). It is a pulsatile biventricular support device implanted after cardiectomy, and is available as 70cc pumps or as the newly manufactured 50cc pumps for smaller patients. Its use has been advocated by Horne et al. especially in Fontan failure with increased pulmonary vascular resistance (PVR) ± ventricular dysfunction (9). Rossano et al. reported the successful implantation of TAH in a 13 year old patient with failing Fontan circulation who survived to transplantation (19).

SV patients present a difficult challenge with regards to optimal configuration of anatomy, unilateral vs. bilateral support, device selection, and cannulation strategies. It is therefore reasonable to suggest that the optimal mechanism for support will vary depending on the etiology of failure. Because of the extreme variability of patients, cardiac anatomy, comorbidities, and mechanism of failure, it is necessary to define ideal management strategies following the three different SV stages: neonatal palliation including Stage I Norwood, superior cavo-pulmonary shunt, and following completion of Fontan circulation.

### After neonatal palliation

During the neonatal period, it is impossible to create a Fontan circulation because of an elevated PVR as well as sizes of the superior vena cava (SVC), inferior vena cava (IVC), and pulmonary arteries. Therefore, a staged approach is utilized which allows the body to adapt progressively to the hemodynamic conditions of each SV stage. Different surgical procedures are used in the neonatal period based on the primary type of CHD. This includes pulmonary artery banding or modified Blalock-Taussig shunt (mBTs) ± Damus-Kaye-Stansel (DKS) anastomosis otherwise known as stage I Norwood procedure. Subsequently, the patient may develop acute or chronic HF. Acute failure should be managed by VA-ECMO support whereas for chronic failure, the best solution may be to progress to the second stage of SV pathway to eliminate a volume-loaded circulation. The use of VA-ECMO support after neonatal palliation with systemic-to-pulmonary artery shunt warrants a balance between systemic and pulmonary perfusion in order to prevent myocardial and systemic ischemia, caused by excessive runoff into the low-resistance pulmonary bed through the shunt.

Early reports of VA-ECMO suggest that outcomes for patients with SV circulation are substantially worse than those with 2-ventricle circulation, and survival to discharge after Stage I Norwood procedure supported with VA-ECMO was only 31% (20, 21). Recent literature indicates an improved outcome of VA- ECMO post Stage I Norwood with a hospital discharge of 50% (22). Laussen et al reviewed 44 patients aged less than 1 year with shunted SV physiology supported with VA-ECMO at Children's Hospital Boston between 1996 and 2005, and reported that the overall survival to discharge in this group of neonates (48%) is comparable to survival reported for all neonatal and pediatric cardiac VA-ECMO in the Extracorporeal Life Support Organization registry (41%) (23).

Ungerleider et al. have advocated the routine use of centrifugal short-term VADs for circulatory support following stage I Norwood for palliation of hypoplastic left heart syndrome (HLHS) (24). Berlin Heart EXCOR has been used in SV failure after neonatal palliation in few cases, mostly as univentricular support with dismal outcome. In 1998, Hetzer et al. reported of a child with HLHS supported with the BHE after stage I Norwood, who died of intracerebral hemorrhage (18). Brancaccio et al. described another child who received a UVAD BHE following a mBTs, who eventually died of a thrombo-embolic event in 2013 (25). Lal et al reported a case of a 14 month old boy with failing SV circulation post-SCPC who was supported with the Revolution VAD (Sorin Group, Arvada, CO) connected to BHE cannulae after his SCPC was taken down and replaced with a mBTs for pulmonary blood flow. Subsequently the child was successfully transplanted (26).

Weinstein et al. reviewed the EXCOR Investigational Device Exemption study database and analyzed outcomes of patients with SV supported with BHE. Amongst 26 children, 9 were supported after palliative neonatal surgery with BHE, in which it served as a UVAD in 8 cases and provided biventricular support in one case, with only one surviving to orthotopic heart transplantation (OHTx) (27). De Rita et al. from Newcastle Upon Tyne in UK, analyzed their experience in patients with two ventricle and SV physiology supported with BHE as bridge to transplant/recovery. Two patients were supported after SV palliative neonatal surgery with BHE, as a UVAD and a BiVAD, respectively, and both died while on support (28). Pearce et al. reported a case of a child supported with BHE as a UVAD after pulmonary artery banding followed by a mBTs for neonatal SV palliation, who was successfully transplanted (29). Recently, Gazit et al. advocated the use of BHE cannulas connected to a centrifugal pump as medium-to-long term support after neonatal univentricular palliation. The benefit of this technique is conferred by the improved stability of the BHE cannulae compared with routine ECMO cannulae placement. Seven patients were treated with this novel technique with 3 patients (43%) being discharged home, two after SCPC and one after OHTx (30).

**Figure 1:**
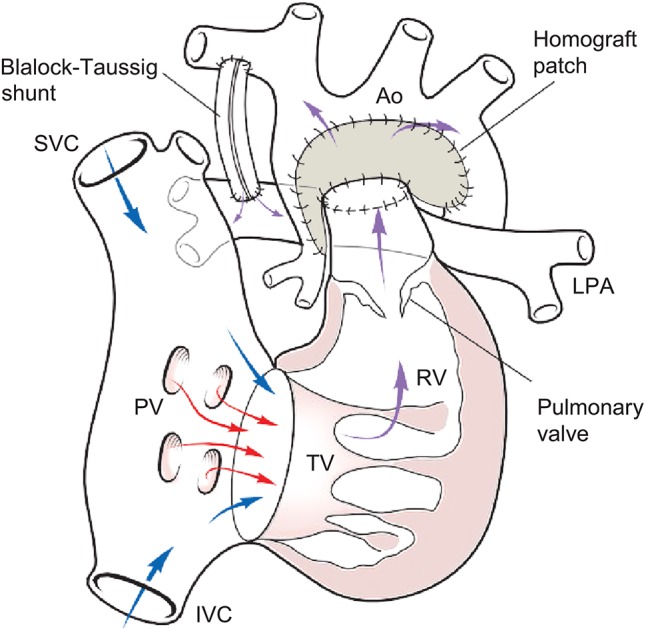
Anatomy following Norwood-mBTs.

**Figure 2:**
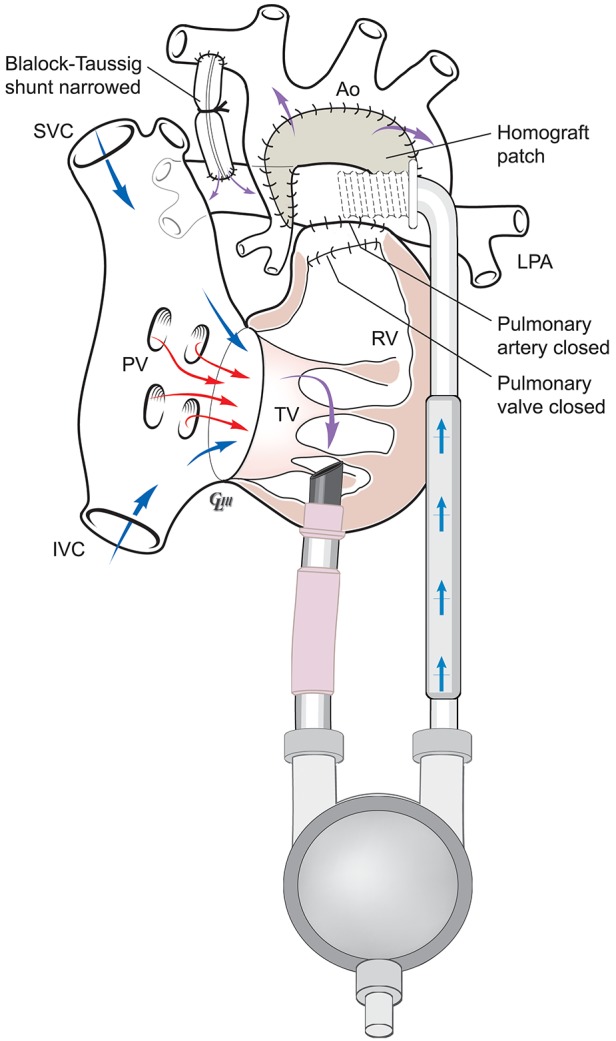
Anatomy following Norwood-mBTs with BHE as a UVAD.

Figure 1 below illustrates the anatomy following a Norwood-mBT shunt operation in a HLHS case. Figure 2 is a diagram of BHE cannulae insertion at this stage, with the inflow cannula inserted in the RV and outflow cannula inserted in the ascending aorta, allowing it to function as a UVAD. The mBTs has been narrowed in order to avoid pulmonary overcirculation.

### After superior cavo-pulmonary shunt

The second operation in the SV pathway involves a superior cavo-pulmonary connection (SCPC) commonly called the “Glenn” shunt, where superior systemic venous return is directed to the pulmonary bed. The main reasons of circulatory failure at this stage are elevated mean pressure in the SCPC, significant atrio-ventricular valve regurgitation, younger age, lower weight, longer bypass time, and re-intubation; whereas, inter-stage mortality was mainly influenced by moderately impaired ventricular function, prolonged hospital stay and poor weight gain (3, 31).

SV patients with SCPC present unique challenges when considering MCS. Since the SVC is connected to the pulmonary artery, decompression of the systemic pumping chamber may not guarantee adequate venous drainage and may result in ongoing venous hypertension, which encourages the development of aberrant veno-venous connections and subsequently worsening cyanosis. Thus the management of circulatory failure at SCPC palliation is mainly medical management, either to recovery or as a bridge to OHTx, with conversion to total cavo-pulmonary circulation or to biventricular repair on rare occasions and subsequent central VA-ECMO or VAD support (14, 32). Particularly, in acute failure it is preferred to use VA-ECMO or VAD, while the therapeutics options for chronic failure are VAD or OHTx.

Recent report on the usage of VA-ECMO in SCPC showed an improved survival to hospital discharge (41%) compared to earlier data, but there was still a high incidence of neurological complications (32). There have been several reports of VAD use with a SCPC palliation as single cases (14, 33, 34). Chu et al. described a 4-year-old with HLHS who developed refractory HF after second stage palliation, was supported with a BHE, and died on postoperative day 13 from bowel necrosis (33). Weinstein et al. described 26 SV patients with a VAD, of which 12 cases were supported after SCPC with BHE (11 as a UVAD and 1 as a BiVAD) and 7 survived to transplantation (27). In addition, De Rita et al. reported 5 patients who were supported after SCPC with BHE (all as a UVAD), and 3 survived to transplant (28). Niebler et al. reported four SV patients palliated with SCPC and supported with BHE in a UVAD configuration with three successfully transplanted (35). Shah et al. reported the implantation of Heartmate II in an adult patient with SCPC who died on support after 261 days (36).

Recent trends in the management of acute univentricular HF with SCPC advocate the use of BHE cannulae connected to a centrifugal pump with an oxygenator and either conversion to a BHE pulsatile pump or maintenance of a continuous flow pump and oxygenator and progress to extubation. Alternatively, the use of an implantable continuous-flow VAD, such as the HVAD Heartware or Heartmate II, may represent a more suitable option due to the ability for discharge to home(37, 38). Regardless of the type of VAD employed, severe hypoxemia related to veno-venous collaterals remains a challenge during VAD support. Performing a concomitant Fontan operation at the time of VAD insertion can alleviate the hypoxemia issue and would provide a more favorable circulation, if candidates are selected appropriately (39).

**Figure 3:**
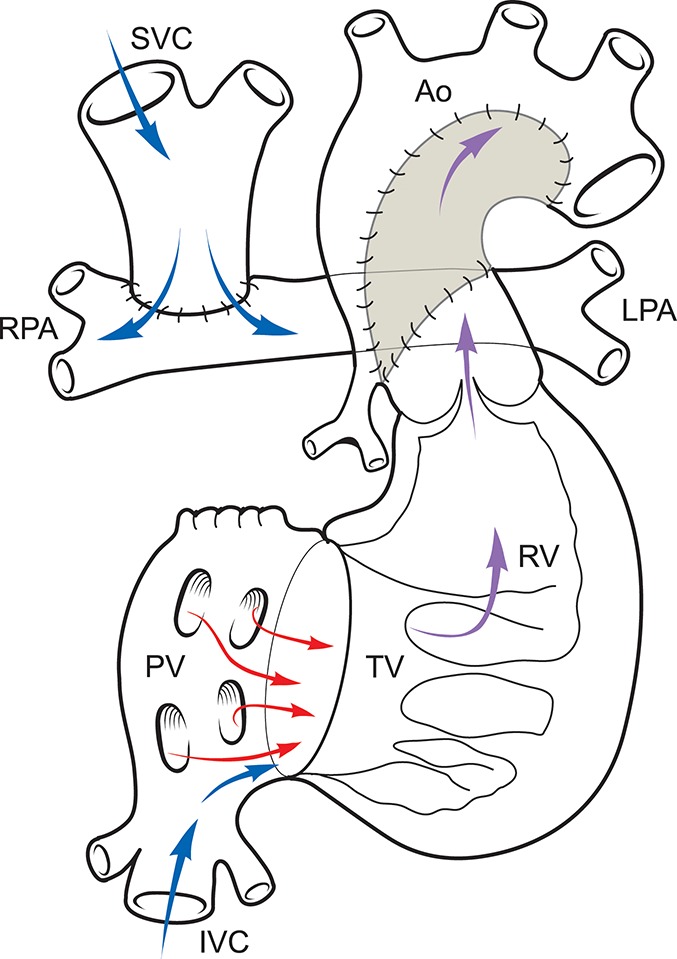
Anatomy following SCPC.

**Figure 4:**
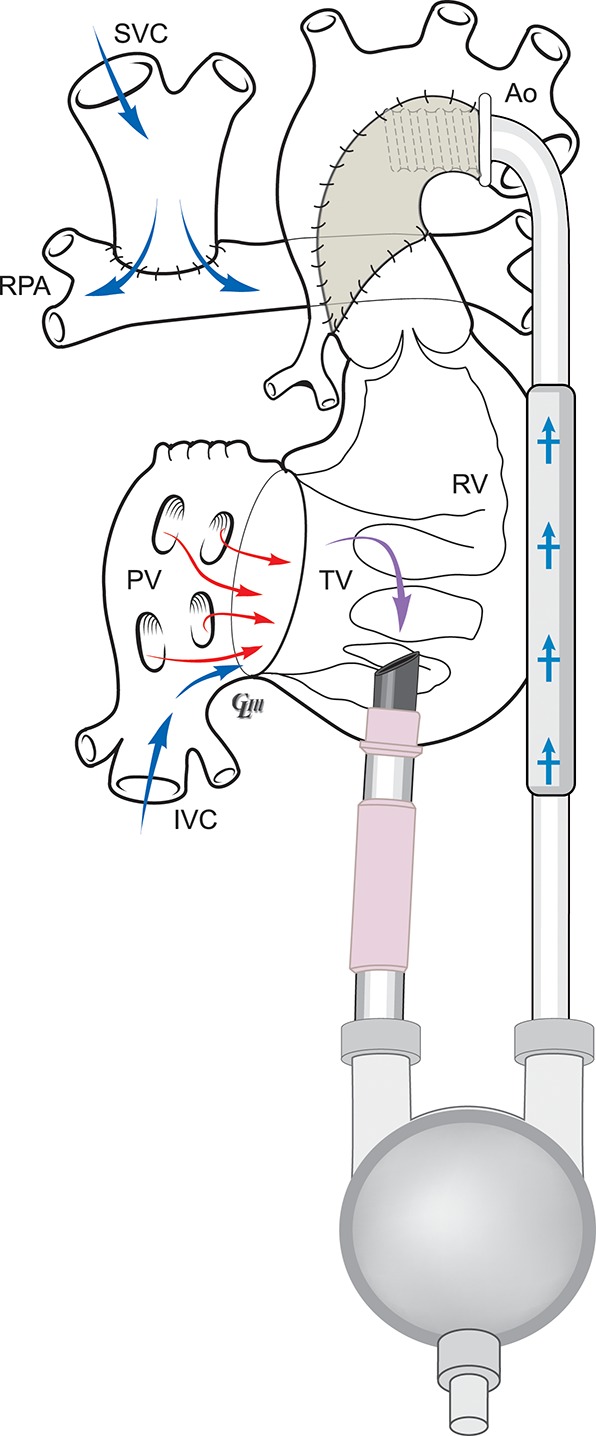
Anatomy of BHE following SCPC as a UVAD.

Figure 3 illustrates the anatomy following a SCPC and routes of systemic and pulmonary blood flow. Figure 4 is a diagram of BHE cannulae insertion with inflow cannula inserted in the RV and outflow cannula inserted in the ascending aorta in this type of anatomy to function as an UVAD.

### After fontan palliation

The inferior systemic venous return is directed to the lung following TCPC. Aim of this stage is that all systemic venous blood flows passively through the pulmonary vascular bed bypassing the SV. The final objective is to achieve normal volume and pressure workload for the SV and nearly normal oxygen saturation of the systemic blood. Prerequisites to a successful TCPC include low PVR and good ventricular function with low end-diastolic pressure (40). After Fontan palliation, various complications such as protein losing enteropathy (PLE) (41), atrial tachyarrhythmias, and progressive ventricular dysfunction can occur, leading to Fontan failure (3). Specifically, up to 40% of patients undergoing TCPC experience HF, and PLE is diagnosed in up to 15% of patients, with a 5-year mortality rate of 50% (3). The mechanism of Fontan failure may be an isolated “pure” pump failure with elevated end-diastolic pressure (L(R)VEDP) with low trans-pulmonary gradient (TPG), sometimes differentiated as a systolic or diastolic, or an isolated “Fontan Circulation” failure, that is more common, and is associated with low L(R)VEDP with high trans-pulmonary gradient (TPG) or a combination of both conditions with elevated L(R)VEDP and elevated TPG (9). Most available conventional interventions are unable to improve the hemodynamic instability of this group of patients and the OHTx may represent the only remaining therapeutic treatment to improve survival and clinical status (42, 43).

Currently, there is no medium or long-term mechanical assist device as bridge-to-transplant or bridge-to-recovery, which has been found suitable for the failing Fontan circulation. VA-ECMO has been used in acute settings in the past, with disappointing results. Rood et al. reported a survival to discharge of 35% in patients with Fontan failure supported with VA-ECMO (44). In the Newcastle Upon Tyne group, 4 children with Fontan failure were supported with VA-ECMO, with three patients surviving to transplantation and one weaning off, after recovery of cardiac function (28). The application of a VAD in the Fontan physiology increases the mean systemic pressure and cardiac output, while providing active drainage of the pulmonary venous atrium thereby effectively decreasing the EDP of the systemic ventricle. Furthermore it increases pulmonary blood flow and systemic venous return, thus ultimately reducing central venous pressure.

**Figure 5:**
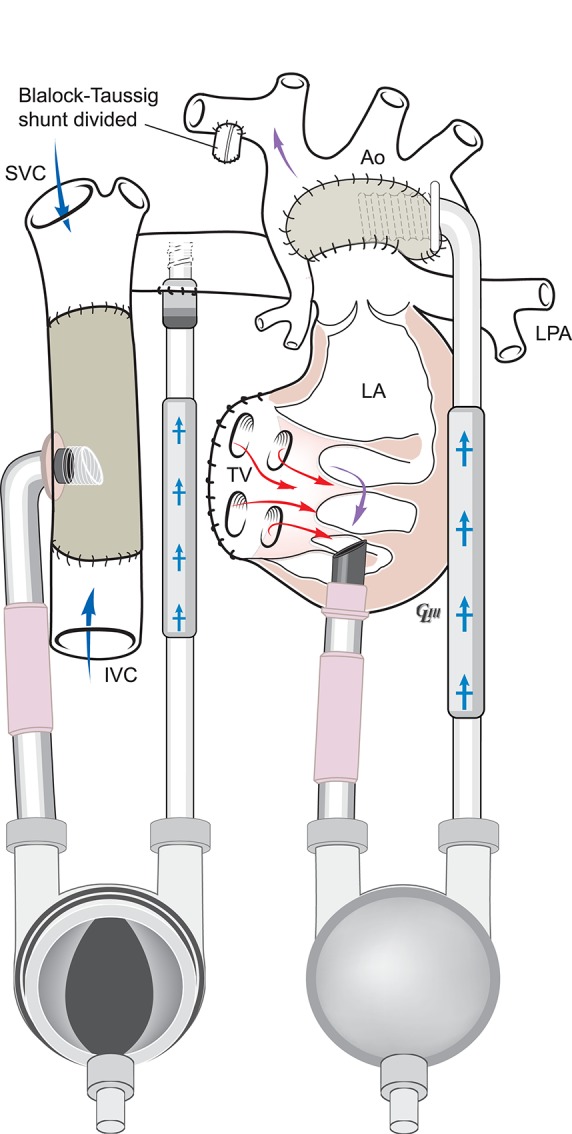
Possible cannulation for BHE BiVAD following TCPC.

BHE has been used in Fontan failure as well, though mainly as a UVAD. In the group described by Weinstein and colleagues, 5 patients with Fontan circulation were supported with BHE as a UVAD and 3 survived to transplantation (27). Sandica et al. and Hoganson et al. published 3 cases of Fontan failure supported with a UVAD BHE and successfully transplanted (37, 45). Nathan et al. reported the placement of a BiVAD BHE in a child with failing Fontan circulation as a bridge to transplantation (46). Similarly, Valeske et al. used a BHE BiVAD as a total artificial heart for “bridge-to-transplantation” in a 19-year-old boy with SV anatomy with failing Fontan circulation and secondary end-stage cardiorespiratory failure (47). Figure 5 illustrates this potential BiVAD set up utilizing a BHE. The inflow cannula is inserted in the extra cardiac Fontan with its associated outflow cannula in the right pulmonary artery for pulmonary circulation, while the inflow cannula is in the RV and outflow cannula in the ascending aorta, for systemic circulatory support.

Prêtre et al. reported a right ventricular assist device (RVAD) with BHE in a 27-year-old patient with the primary diagnosis of tricuspid atresia, extracardiac Fontan and preserved ventricular function, who was eventually transplanted successfully (48). VanderPluym et al. described a case of a failing Fontan, being supported to transplantation with a UVAD BHE after reconversion to SCPA, in a 3-year-old child (10). Intracorporeal devices have been used in failing TCPC as well. Axial flow pumps usage in failing Fontan patients has been reported by Frazier et al. (HeartMate I in 2005 and HeartMate II in 2015), Morales et al. and Shah et al. (HeartMate II) whereas reports citing centrifugal pumps (HeartWare) were from Miera et al., Niebler et al., and Arnaoutakis (11, 36, 49–53).

Few other devices have been attempted in the failing Fontan circulation including the Thoratec Paracorporeal Pulsatile VAD (Thoratec Corp, Pleasanton, CA), which was used in a case, reported by Newcomb et al. from Melbourne who survived to transplantation and in another case, reported by Arnaoutakis et al. who did not survived to heart transplantation (53, 54). Russo et al. described a case of a failing Fontan who was successfully transplanted after been bridged with a centrifugal VAD followed by Thoratec Intracorporeal Pulsatile VAD (55). An alternative strategy is the use of TAH as bridge to transplantation (53). So far, there has been limited experience in this for SV patients, but we are expecting the number of implants to grow in the future, considering the availability of smaller pumps and increasing clinical applications.

## Discussion

The management of SV failure is a very difficult task for surgeons and physicians, as it involves a complex underlying pathophysiology often confounded by other medical and surgical issues. The mode, timing, and mechanism of SV failure as well the patient size are equally important factors in deciding the management strategy. The algorithm proposed by Horne et al. is a valid attempt to rationalize the care in SV failure, but frequently an individualized approach is warranted in view of other factors (9).

End-organ failure, specifically renal and hepatic, often develop due to long-standing suboptimal circulation. This constitutes a considerable problem when dealing with SV physiology and also plays an important role in candidacy for future transplantation. The ability to preserve the liver's synthetic and metabolic functions is paramount for the success of cardiac transplantation, especially in patients with Fontan failure.

Short-term device use for initial MCS support is often necessary in SV patients with acute HF. This is beneficial as it can be used as a bridge to decision in order to allow full assessment of each major organ system. However, the associated substantial neurological risks are an important consideration (24, 32). Therefore, assessment of the central nervous system (CNS) is critical prior to implantation of a more durable MCS in cases where VA-ECMO is used for acute deteriorations.

Although it has been demonstrated that using more than one modality of MCS does not increase the neurological risk, this may not be the case with the SV population (53). Thus, it may be beneficial to employ a more definitive type of MCS from the beginning in SV patients with chronic HF. This would avoid chest re-explorations, change of cannulae, and multiple runs of cardio-pulmonary bypass. The usage of BHE cannulae for centrifugal VAD with or without an oxygenator is a good initial compromise, which allow the flexibility to change to pulsatile BHE pumps and reconversion to non-pulsatile flow if necessary. This approach, mainly employed in small children, simplifies the surgical issues, as these cases are already complicated by multiple sternotomies, presence of allograft or synthetic materials, pacing system, and risk of bleeding due to concomitant anticoagulation or anti-platelets treatment. Furthermore, the vast majority of these complicated patients stay in intensive care units for substantial period of time. This results in the continuous need for vascular access for monitoring in the setting of limited available arterial and venous sites due to possible multiple cardiac catheterizations.

Recently there has been an increasing number of mechanical devices available for the surgeons, however MCS in SV failure is centered only on few of those, as reported by the literature (9, 30, 53). Centrifugal pumps can be used with an oxygenator as VA-ECMO or without as a VAD, either as a UVAD leaving the mBTs, the SCPC, or the TCPC as a source of pulmonary blood flow, or as a BiVAD after recreating a venous reservoir for systemic venous drainage (9). In the same way, the BHE has been used as a UVAD in the vast majority of cases or as BiVAD. The most widely used continuous flow intracorporeal devices are the HeartMate II axial flow pump and the HeartWare centrifugal pump.

While in the past, the choice between these devices was mainly related to the surgeon's and institution's preferences, this may be changing nowadays. Currently, the choice is dependent on the mode of failure in relation to the mechanism of each device, particularly considering their unloading characteristics. The TAH opens an interesting perspective for the future as it can provide biventricular support with high pump output. Given the ongoing miniaturization, the TAH system will become available even in smaller patients. However, it involves extensive surgery, needs the creation of a venous reservoir for the systemic return, and can be further complicated by anatomical variation as situs inversus, dextrocardia, and isomeric hearts. Thus, it is clear that the MCS support for SV population has not reached the level of sophistication compared to that for normally structured hearts. More clinical experience as well as academic studies are necessary for further improvement in this challenging population.

## Conclusion

Literature has shown that it is possible to support patients with SV failure using MCS, however the results of such usage are mixed, based on the underlying anatomy. Moreover, the outcomes of MCS in stage I patients are far from optimal. Although cardiac transplantation represents the ultimate goal for SV failure patients, the lack of suitable donors remains a considerable problem. The understanding of the underlying SV pathophysiology and its interaction with MCS devices continues to evolve. Together with the development of new devices, we hope to provide MCS with medium and long-term durability for these patients, with comparable results to those with two-ventricle physiology.

## Author contributions

MG collected articles, wrote and revised manuscript. RS collected articles, revised manuscript. SJ collected articles, revised manuscript. GP collected articles, wrote manuscript. IA collected articles, wrote manuscript.

### Conflict of interest statement

IA serves as a consultant and proctor for Berlin Heart, Inc., and Heartware, Inc. IA receives a research grant from Abiomed, Inc. The remaining authors declare that the research was conducted in the absence of any commercial or financial relationships that could be construed as a potential conflict of interest.

## Abbreviations

BHE: Berlin Heart EXCOR
BiVAD: biventricular assist device
CHD: congenital heart disease
CNS: central nervous system
DKS: Damus-Kaye Stansel
EDP: end-diastolic pressure
HD: hemodialysis
HF: heart failure
HLHS: hypoplastic left heart syndrome
IVC: inferior vena cava
mBTs: modified Blalock-Taussig shunt
MCS: mechanical circulatory support
OHTx: orthotopic heart transplantation
PLE: protein losing enteropathy
PVR: pulmonary vascular resistance
RVAD: right ventricular assist device
SCPC: superior cavopulmonary connection
SV: single ventricle
SVC: superior vena cava
TAH: total artificial heart
TCPC: total cavopulmonary connection
TPG: transpulmonary gradient
UVAD: univenticular assist device
VA-ECMO: veno-arterial extracorporeal membrane oxygenation
VAD: ventricular assist device.

## References

[1] Fontan F Baudet E. Surgical repair of tricuspid atresia. Thorax (1971) 26:240–8. 10.1136/thx.26.3.240 5089489 PMC1019078

[2] Khairy P Poirier N Mercier L. Univentricular Heart. Circulation (2007) 115:800–12. 10.1161/circulationaha.105.592378 17296869

[3] Ghanayem NS Berger S Tweddell JS. Medical management of the failing Fontan. Pediatric Cardiol. (2007) 28:465–71. 10.1007/s00246-007-9007-0 17763892

[4] Stanford W Armstrong RG Cline RE King TD. Right atrium-pulmonary artery allograft for correction of tricuspid atresia. J Thorac Cardiovasc Surg. (1973) 66:105–11. 4577104

[5] Kreutzer GO Vargas FJ Schlichter AJ Laura JP Suarez JC Coronel AR. Atriopulmonary anastomosis. J Thorac Cardiovasc Surg. (1982) 83:427–36. 7062754

[6] de Leval MR Kilner P Gewillig M Bull C. Total cavopulmonary connection: a logical alternative to atriopulmonary connection for complex Fontan operations. Experimental studies and early clinical experience. J Thorac Cardiovasc Surg. (1988) 96:682–95. 3184963

[7] Marcelletti C Corno A Giannico S Marino B. Inferior vena cava-pulmonary artery extracardiac conduit. A new form of right heart bypass. J Thorac Cardiovasc Surg. (1990) 100:228–32. 2143549

[8] Rossano JW Goldberg DJ Mott AR Ravishankar C Gaynor JW Falkensammer C Hospitalizations in single ventricle patients in the United States: prevalence and outcomes from 2000 to 2006. J Am Coll Cardiol. (2012) 59:A180 10.1016/S0735-1097(12)60746-3

[9] Horne D Conway J Rebeyka IM Buchholz H. Mechanical circulatory support in univentricular hearts: current management. Semin Thorac Cardiovasc Surg Pediatr Card Surg Ann. (2015) 18:17–24. 10.1053/j.pcsu.2015.02.002 25939838

[10] Vanderpluym CJ Rebeyka IM Ross DB Buchholz H. The use of ventricular assist devices in pediatric patients with univentricular hearts. J Thorac Cardiovasc Surg. (2011) 141:588–90. 10.1016/j.jtcvs.2010.06.038 20692001

[11] Morales DL Adachi I Heinle JS Fraser CD. A new era: use of an intracorporeal systemic ventricular assist device to support a patient with a failing Fontan circulation. J Thorac Cardiovasc Surg. (2011) 142:e138–40. 10.1016/j.jtcvs.2011.05.018 21762934

[12] Rossano JW Woods RK Berger S Gaynor JW Ghanayem N Morales DL. Mechanical support as failure intervention in patients with cavopulmonary shunts (MFICS): rationale and aims of a new registry of mechanical circulatory support in single ventricle patients. Congenit Heart Dis. (2013) 8:182–6. 10.1111/chd.12053 23510301

[13] Humpl T Furness S Gruenwald C Hyslop C Arsdell GV. The berlin heart EXCOR pediatrics-the SickKids experience 2004-2008. Artif Organs (2010) 34:1082–6. 10.1111/j.1525-1594.2009.00990.x 20545666

[14] Irving CA Cassidy JV Kirk RC Griselli M Hasan A Crossland DS. Successful bridge to transplant with the Berlin Heart after cavopulmonary Shunt. J Heart Lung Trans. (2009) 28:399–401. 10.1016/j.healun.2008.12.009 19332269

[15] Feingold B Bowman P Zeevi A Girnita AL Quivers ES Miller SA. Survival in allosensitized children after listing for cardiac transplantation. J Heart Lung Trans. (2007) 26:565–71. 10.1016/j.healun.2007.03.015 17543778

[16] Jefferies JL Morales DL. Mechanical circulatory support in children: bridge to transplant versus recovery. Curr Heart Fail Rep. (2012) 9:236–43. 10.1007/s11897-012-0103-y 22805892

[17] Miera O Schmitt KR Delmo-Walter E Ovroutski S Hetzer R Berger F. Pump size of Berlin Heart EXCOR pediatric device influences clinical outcome in children. J Heart Lung Trans. (2014) 33:816–21. 10.1016/j.healun.2014.03.007 24836553

[18] Hetzer R Loebe M Potapov EV Weng Y Stiller B Hennig E. Circulatory support with pneumatic paracorporeal ventricular assist device in infants and children. Ann Thorac Surg. (1998) 66:1498–505. 10.1016/s0003-4975(98)00914-x 9875742

[19] Rossano JW Goldberg DJ Fuller S Ravishankar C Montenegro LM Gaynor JW. Successful use of the total artificial heart in the failing Fontan circulation. Ann Thorac Surg. (2014) 97:1438–40. 10.1016/j.athoracsur.2013.06.120 24694426

[20] Kulik TJ Moler FW Palmisano JM Custer JR Mosca RS Bove EL. Outcome- associated factors in pediatric patients treated with extracorporeal membrane oxygenator after cardiac surgery. Circulation (1996) 94:II63–68. 8901721

[21] Sherwin ED Gauvreau K Scheurer MA Rycus PT Salvin JW Almodovar MC. Extracorporeal membrane oxygenation after stage 1 palliation for hypoplastic left heart syndrome. J Thorac Cardiovasc Surg. (2012) 144:1337–43. 10.1016/j.jtcvs.2012.03.035 22503203

[22] Pizarro C Davis D Healy R Kerins P Norwood W. Is there a role for extracorporeal life support after stage I Norwood? Eur J of Cardiothorac Surg. (2001) 19: 294–301. 10.1016/s1010-7940(01)00575-9 11251269

[23] Allan CK Thiagarajan RR Nido PJ Roth SJ Almodovar MC Laussen PC. Indication for initiation of mechanical circulatory support impacts survival of infants with shunted single-ventricle circulation supported with extracorporeal membrane oxygenation. J Thorac Cardiovasc Surg. (2007) 133:660–67. 10.1016/j.jtcvs.2006.11.013 17320562

[24] Ungerleider RM Shen I Yeh T Schultz J Butler R Silberbach M. Routine mechanical ventricular assist following the Norwood procedure–improved neurologic outcome and excellent hospital survival. Ann Thorac Surg. (2004) 77:18–22. 10.1016/s0003-4975(03)01365-1 14726027

[25] Brancaccio G Gandolfo F Carotti A Amodeo A. Ventricular assist device in univentricular heart physiology. Interact Cardiovasc Thorac Surg. (2013) 16:568–9. 10.1093/icvts/ivs559 23322095 PMC3598047

[26] Lal AK Chen S Maeda K Mccammond A Rosenthal DN Reinhartz O. Successful bridge to transplant with a continuous flow ventricular assist device in a single ventricle patient with an aortopulmonary shunt. ASAIO J. (2014) 60:119–21. 10.1097/mat.0000000000000007 24270233

[27] Weinstein S Bello R Pizarro C Fynn-Thompson F Kirklin J Guleserian K. The use of the Berlin Heart EXCOR in patients with functional single ventricle. J Thorac Cardiovasc Surg. (2014) 147:697–705. 10.1016/j.jtcvs.2013.10.030 24290716

[28] Rita FD Hasan A Haynes S Crossland D Kirk R Ferguson L Mechanical cardiac support in children with congenital heart disease with intention to bridge to heart transplantation. Eur J Cardiothorac Surg. (2014) 46:656–62. 10.1093/ejcts/ezu039 24578411

[29] Pearce FB Kirklin JK Holman WL Barrett CS Romp RL Lau YR. Successful cardiac transplant after Berlin Heart bridge in a single ventricle heart: use of aortopulmonary shunt as a supplementary source of pulmonary blood flow. J Thorac Cardiovasc Surg. (2009) 137:e40–2. 10.1016/j.jtcvs.2008.02.044 19154881

[30] Gazit AZ Petrucci O Manning P Shepard M Baltagi S Simpson K. A novel surgical approach to mechanical circulatory support in univentricular infants. Ann Thorac Surg. (2017) 104:1630–6. 10.1016/j.athoracsur.2017.04.023 28720202

[31] Nichay NR Gorbatykh YN Kornilov IA Soynov IA Kulyabin YY Gorbatykh AV. Risk factors for unfavorable outcomes after bidirectional cavopulmonary anastomosis. World J Pediatr Congenit Heart Surg. (2017) 8:575–83. 10.1177/2150135117728505 28901234

[32] Jolley M Thiagarajan RR Barrett CS Salvin JW Cooper DS Rycus PT. Extracorporeal membrane oxygenation in patients undergoing superior cavopulmonary anastomosis. J Thorac Cardiovasc Surg. (2014) 148:1512–8. 10.1016/j.jtcvs.2014.04.028 24951018

[33] Chu MW Sharma K Tchervenkov CI Jutras LF Lavoie J Shemie SD. Berlin Heart ventricular assist device in a child with hypoplastic left heart syndrome. Ann Thorac Surg. (2007) 83:1179–81. 10.1016/j.athoracsur.2006.08.020 17307489

[34] Mackling T Shah T Dimas V Guleserian K Sharma M Forbess J. Management of single-ventricle patients with Berlin Heart EXCOR ventricular assist device: single-center experience. Artif Organs. (2012) 36:555–9. 10.1111/j.1525-1594.2011.01403.x 22236151

[35] Niebler RA Shah TK Mitchell ME Woods RK Zangwill SD Tweddell JS. Ventricular assist device in single-ventricle heart disease and a superior cavopulmonary anastomosis. Artif Organs. (2015) 40:180–4. 10.1111/aor.12531 26147841

[36] Shah NR Lam WW Rodriguez FH Ermis PR Simpson L Frazier O. Clinical outcomes after ventricular assist device implantation in adults with complex congenital heart disease. J Heart Lung Transplant. (2013) 32:615–20. 10.1016/j.healun.2013.03.003 23540399

[37] Sandica E Blanz U Mime LB Schultz-Kaizler U Kececioglu D Haas N. Long-term mechanical circulatory support in pediatric patients. Artif Organs. (2015) 40:225–32. 10.1111/aor.12552 26411865

[38] Adachi I Jeewa A Burki S Mckenzie ED Fraser CD. Outpatient management of a child with bidirectional Glenn shunts supported with implantable continuous-flow ventricular assist device. J Heart Lung Transplant. (2016) 35:688–90. 10.1016/j.healun.2016.01.1218 27056613

[39] Adachi I Williams E Jeewa A Elias B Mckenzie ED. Mechanically assisted Fontan completion: a new approach for the failing Glenn circulation due to isolated ventricular dysfunction. J Heart Lung Transplant (2016) 35:1380–1. 10.1016/j.healun.2016.09.011 27836026

[40] Border WL Syed AU Michelfelder EC Khoury P Uzark KC Manning PB. Impaired systemic ventricular relaxation affects postoperative short-term outcome in Fontan patients. J Thorac Cardiovasc Surg. (2003) 126:1760–4. 10.1016/j.jtcvs.2003.06.006 14688684

[41] Michielon G Parisi F Carlo DD Squitieri C Carotti A Buratta M. Orthotopic heart transplantation for failing single ventricle physiology. Eur J Cardiothorac Surg. (2003) 24:502–10. 10.1016/s1010-7940(03)00342-7 14500066

[42] Chakrabarti S. Acquired combined immunodeficiency associated with protein losing enteropathy complicating Fontan operation. Heart (2003) 89:1130–1. 10.1136/heart.89.10.1130 12975395 PMC1767867

[43] Carey JA Hamilton JL Hilton CJ Dark JH Forty J Parry G. Orthotopic cardiac transplantation for the failing Fontan circulation. Eur J Cardiothorac Surg. (1998) 14:7–14. 10.1016/s1010-7940(98)00130-4 9726608

[44] Rood KL Teele SA Barrett CS Salvin JW Rycus PT Fynn-Thompson F. Extracorporeal membrane oxygenation support after the Fontan operation. J Thorac Cardiovasc Surg. (2011) 142:504–10. 10.1016/j.jtcvs.2010.11.050 21353253

[45] Hoganson DM Boston US Gazit AZ Canter CE Eghtesady P. Successful bridge through transplantation with berlin heart ventricular assist device in a child with failing fontan. Ann Thorac Surg. (2015) 99:707–9. 10.1016/j.athoracsur.2014.04.064 25639417

[46] Nathan M Baird C Fynn-Thompson F Almond C Thiagarajan R. Successful implantation of a Berlin heart biventricular assist device in a failing single ventricle. J Thorac Cardiovasc Surg. (2006) 131:1407–8. 10.1016/j.jtcvs.2006.02.015 16733184

[47] Valeske K Yerebakan C Mueller M Akintuerk H. Urgent implantation of the Berlin Heart Excor biventricular assist device as a total artificial heart in a patient with single ventricle circulation. J Thorac Cardiovasc Surg. (2014) 147:1712–4. 10.1016/j.jtcvs.2014.01.012 24521952

[48] Prêtre R Häussler A Bettex D Genoni M. Right-sided univentricular cardiac assistance in a failing Fontan circulation. Ann Thorac Surg. (2008) 86:1018–20. 10.1016/j.athoracsur.2008.03.003 18721610

[49] Frazier OH Gregoric ID Messner GN. Total circulatory support with an LVAD in an adolescent with a previous Fontan procedure. Texas Heart Inst J. (2005) 32:402–4. 16392230 PMC1336720

[50] Jabbar AA Franklin WJ Simpson L Civitello AB Delgado RM Frazier O. Improved systemic saturation after ventricular assist device implantation in a patient with decompensated dextro-transposition of the great arteries after the Fontan procedure. Texas Heart Inst J. (2015) 42:40–3. 10.14503/thij-13-3374 25873797 PMC4378042

[51] Niebler RA Ghanayem NS Shah TK Bobke AD Zangwill S Brosig C. Use of a HeartWare ventricular assist device in a patient with failed Fontan circulation. Ann Thorac Surg. (2014) 97:e115–6. 10.1016/j.athoracsur.2013.11.075 24694452

[52] Miera O Potapov EV Redlin M Stepanenko A Berger F Hetzer R. First experiences with the HeartWare ventricular assist system in children. Ann Thorac Surg. (2011) 91:1256–60. 10.1016/j.athoracsur.2010.12.013 21440155

[53] Arnaoutakis GJ Blitzer D Fuller S Eckhauser AW Montenegro LM Rossano JW. Mechanical circulatory support as bridge to transplantation for the failing single ventricle. Ann Thorac Surg. (2017) 103:193–7. 10.1016/j.athoracsur.2016.05.015 27424467

[54] Newcomb AE Negri JC Brizard CP D'Udekem Y. Successful left ventricular assist device bridge to transplantation after failure of a fontan revision. J Heart Lung Trans. (2006) 25:365–7. 10.1016/j.healun.2005.05.022 16507435

[55] Russo P Wheeler A Russo J Tobias JD. Use of a ventricular assist device as a bridge to transplantation in a patient with single ventricle physiology and total cavopulmonary anastomosis. Paediatr Anaesth (2008) 18:320–4. 10.1111/j.1460-9592.2008.02435.x 18315638

